# Malignant Pleural Mesothelioma, Biphasic Type: An Unusual and Insidious Case of Rapidly Progressive Small Blue Cell Tumor

**DOI:** 10.7759/cureus.2749

**Published:** 2018-06-06

**Authors:** Carlos Salazar, NELSON KANTER MD, Amer Abboud

**Affiliations:** 1 Department of Internal Medicine, Louis A. Weiss Memorial Hospital, Chicago, USA; 2 Department of Pulmonary and Critical Care Medicine, Louis A. Weiss Memorial Hospital, Chicago, USA; 3 Department of Pathology, Louis A. Weiss Memorial Hospital, Chicago, USA

**Keywords:** malignant pleural mesothelioma, asbestos

## Abstract

Malignant pleural mesothelioma (MPM) is a rare neoplasm. It predominantly affects elderly individuals aged over 70 years presenting with a unilateral pleural tumor usually associated with previous asbestos exposure. The respiratory symptoms are associated with ipsilateral pleural involvement with concomitant pleural effusions. The diagnosis of MPM is established by the morphologic and immunohistochemical features of a cytologic specimen. MPM can present as three histologic subtypes: epithelioid, sarcomatoid, or biphasic. We present a case of an 85-year-old Caucasian female with a history of occupational asbestos exposure. She complained of 1-week history of progressive sharp right flank and scapular pain with mild shortness of breath, dry cough and pleuritic chest pain. CT of the chest showed a large loculated right pleural effusion with adjacent pleural thickening. CT abdomen and pelvis was negative for other neoplastic findings. CT-guided core biopsy of the right pleural-based mass was positive for a spindle to plasmacytoid small blue cell tumor. An extensive immunohistochemical panel was non-specific. A focal OSCAR keratin and WT-1 expression in the absence of carcinoma markers, a malignant mesothelioma, biphasic type was diagnosed. Further workup with PET-CT and cytotoxic chemotherapy combined with immunotherapy or tyrosine kinase inhibitors was recommended by oncology. The patient refused further imaging and treatment, and palliative care was consulted.

## Introduction

Malignant pleural mesothelioma (MPM) is a rare and insidious neoplasm. It predominantly affects individuals aged over 70 years who present with a unilateral pleural tumor usually associated with previous asbestos exposure. The latency period for mesothelioma after initial exposure to asbestos is typically longer than 30 years; therefore, screening for MPM is challenging [1-2]. It is estimated that 43,000 patients die from this disease each year. It has also been estimated that there are 10,000 mesothelioma cases annually in North America, Western Europe, Australia, and Japan combined [3]. Initial symptoms of MPM are non-specific and can be misleading, generally shortness of breath, chest pain, weight loss, and fatigue [4]. Respiratory symptoms are associated with ipsilateral pleural involvement with concomitant pleural effusions and intrathoracic spread. Bone and neuropathic pain may occur if there is involvement of the neural intercostal, paravertebral, or brachial plexus structures [5]. Pleural mesothelioma is diagnosed by the morphologic and immunohistochemical features of a cytologic or surgical specimen. [6] MPM can present as three histologic subtypes: epithelioid, sarcomatoid, or biphasic or mixed. The International Mesothelioma Interest Group (IMIG) recommends using a panel of immunoreactive and non-immunoreactive markers to establish the diagnosis; however, when there are discordant findings, additional markers should be used. [7] It is important to rule out other types of malignancies, for example the WT1 marker is expressed in most epithelioid mesotheliomas but absent in squamous cell carcinomas, making this the best positive mesothelioma marker for discriminating between those malignancies [8]. The biphasic or mixed subtype can be found in 30% of cases and is characterized by a mixture of epithelioid and sarcomatoid or spindle features [9]. Each histologic type must make up at least 10% of the neoplasm to meet the diagnostic criteria. Carcinosarcomas, biphasic pulmonary blastoma or biphasic synovial sarcoma may also exhibit a biphasic or mixed pattern and should be ruled out [10]. The standard treatment for MPM is cisplatin plus pemetrexed chemotherapy. The addition of bevacizumab, an anti-vascular endothelial growth factor antibody, combined with cisplatin plus pemetrexed has shown some promising results in recent studies [11]. We present a case of an elderly woman with occupational asbestos exposure presenting with nonspecific unilateral flank and chest pain due to a large pleural mass with effusion. 

## Case presentation

**Figure 1:**
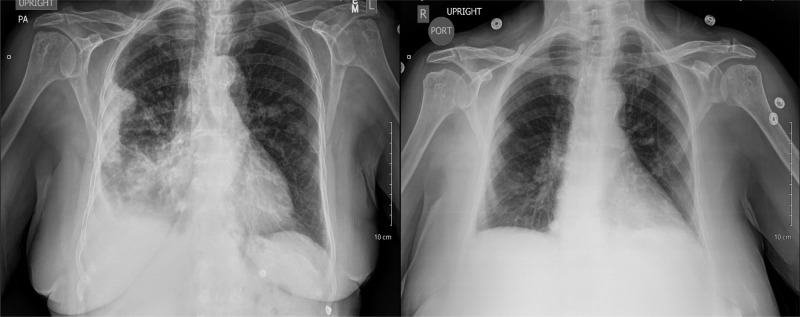
Posteroanterior and portable chest X-ray (Left figure) Posteroanterior chest X-ray showing dense peripheral right lung pleural-based opacity with blunting of the right costophrenic angle. Interstitial prominence of the right lung is present with right lower lung opacity. Nodular opacities are seen in the left midlung. (Right figure) Portable chest X-ray 2 months prior to the image at the left, showing no focal airspace opacity. A calcified nodule is seen at the right cardiophrenic junction which is unchanged compared to previous images.

An 85-year-old Caucasian female presented to our institution complaining of a one-week history of progressive sharp right flank and scapular pain with mild shortness of breath, dry cough, and pleuritic chest pain exacerbated with deep inspiration. The patient denied constitutional symptoms, nausea, vomiting, diarrhea, or constipation. The past medical history was significant for essential hypertension, hypercholesterolemia, rheumatoid arthritis, and asthma; and surgical history significant for uterine benign tumor removal and unilateral oophorectomy 40 years prior. The patient was allergic to iodinated contrast media. The social history included 15 years of occupational asbestos exposure, southeastern European descent, and nonsmoker. Vital signs were significant for uncontrolled arterial hypertension and oxygen saturation of 93% on room air. Physical examination showed no acute respiratory distress, mild bibasilar crackles greater on the right side, diffuse abdominal tenderness and right costovertebal angle tenderness. Initial laboratory studies revealed normocytic normochromic anemia and arterial blood gas analysis consistent with acute respiratory alkalosis. The comprehensive metabolic panel, lipase, troponins and EKG were unremarkable. The chest X-ray showed a dense peripheral right lung pleural-based opacity and blunting of the right costophrenic angle and multiple nodular opacities in the left midlung. Findings were new compared to previous imaging test done two months prior to presentation (Figure 1). The patient had multiple previous visits to the emergency department with similar complaints and negative workups. 

**Figure 2:**
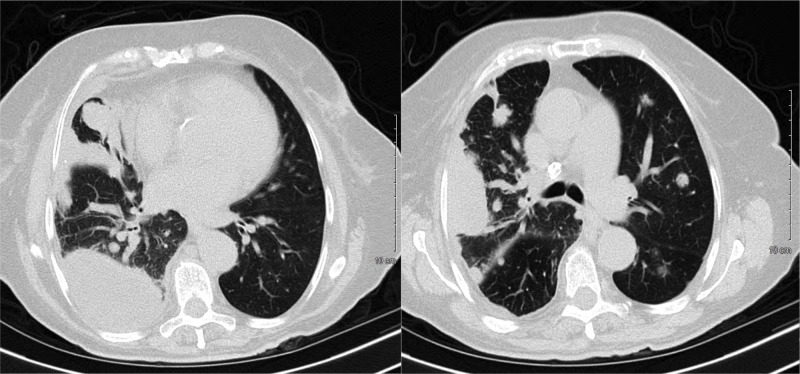
CT of the chest without contrast (Left figure) Large loculated right pleural effusion within the right lung base with adjacent pleural thickening anteriorly, multiple small medium and large size nodular masses scattered throughout both lung fields, especially involving the right lung and right pleura. (Right figure) Large area of nodular consolidation and mass is seen along the right lateral chest wall midportion measuring up to 5.5 x 3 cm in length and thickness. Multiple left lung nodules vary in size from 1 cm up to 2 cm in size.

CT chest without contrast showed a large loculated right pleural effusion with adjacent pleural thickening. In addition, there were multiple masses throughout both lung fields, greater on the right (Figure 2). CT abdomen and pelvis were negative for other neoplastic findings. A flexible bronchoscopy was performed and showed only tracheal and right lung bronchial edema. Bronchial washing and bronchial biopsies were negative for neoplastic or infectious processes. A CT-guided core biopsy of the right pleural-based mass was performed. At the same time, 300 mL of red fluid was obtained from an ultrasound-guided thoracentesis from the right pleural effusion. Analysis showed an exudative fluid with 60,000 RBC/UL with lymphocytic predominance. The cytologic analysis of the pleural fluid was negative for malignancy. 

The initial pathologic report from the pleural mass was positive for malignancy, exhibiting a spindle to plasmacytoid small blue cell tumor. The cellular smear showed numerous non-cohesive to loosely cohesive plasmacytoid cells. Immunohistochemical stains done in our institution were diffusely positive for Vimentin and focally positive for CD138. Given the CD138 focal positivity, the possibility of a hematolymphoid malignancy or plasma cell disorder was considered; however, workup was essentially negative. The samples were sent to Mayo Clinic Medical Laboratory, Rochester, MN for confirmation. The external pathology report revealed neoplastic cells that were diffusely positive for Vimentin, focally positive for OSCAR keratin, WT-1, CD138 and CAM5.2, and rarely positive for ALK. All other markers tested were essentially negative (Table 1). 

**Table 1:** Immunoperoxidase stain report Paraffin sections of the right lung mass using antibodies directed against specific antigens. (+++) diffusely positive, (++) focally positive, (+) rarely positive, (-) negative.

Antigens and Results			
Vimentin	+++	p63	-
WT1	++	MOC-31	-
OSCAR keratin	++	ER	-
CD138	++	S100	-
CAM 5.2	++	HMB-45	-
ALK	+	D2-40	-
CD45	-	PAX5	-
CD34	-	CK7	-
CD30	-	CK20	-
CD99	-	MART-1	-
CK5/6	-	Synaptophysin	-
SMA	-	Chromogranin	-
CD56	-	TTF-1	-
SOX10	-	Keratin AE1/AE3	-
Myogenin	-	Calretinin	-
CK5/6	-	MUM-1	-

**Figure 3:**
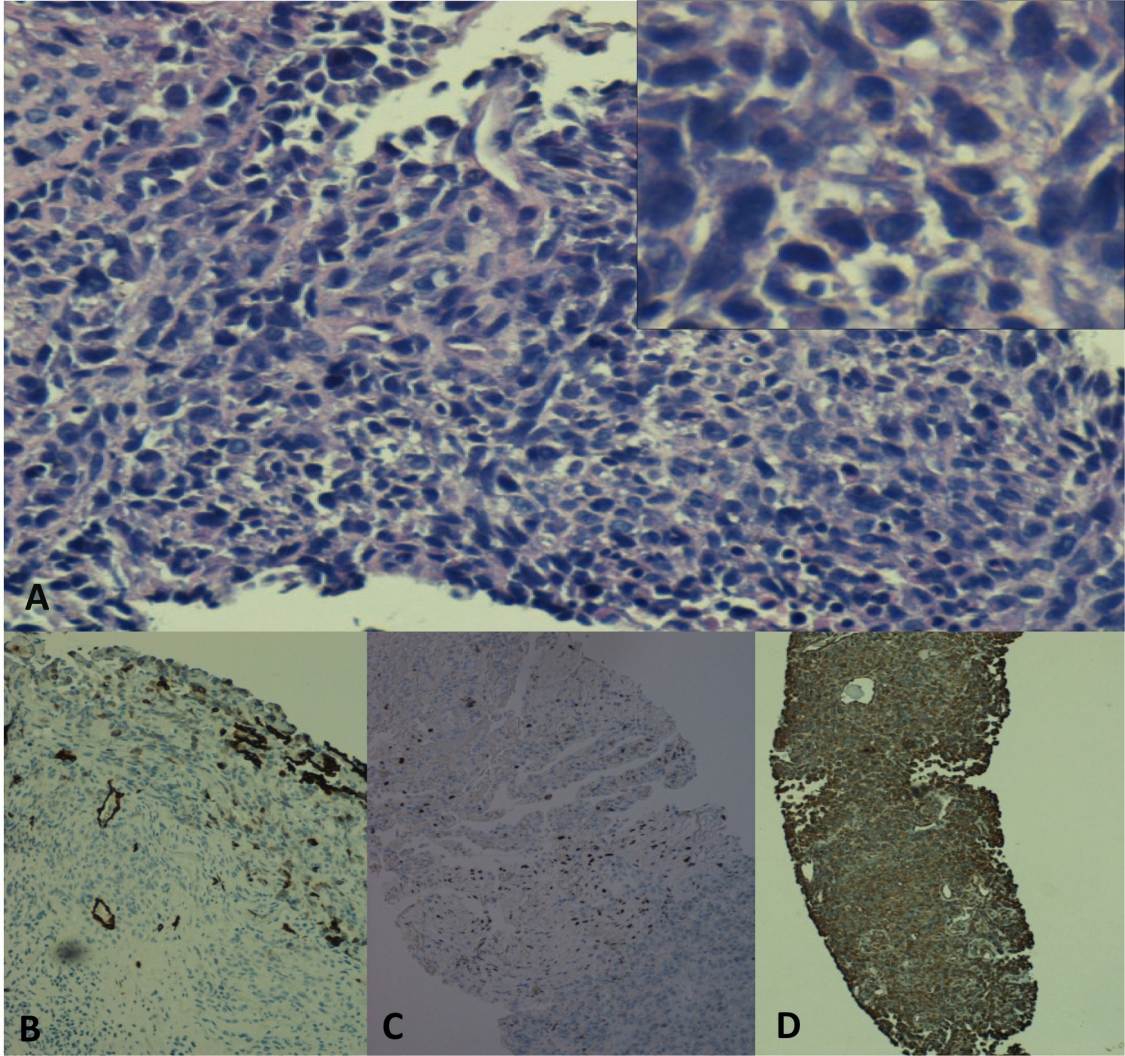
Histological examination and immunohistochemical stains Histological examination (A) Hematoxylin and eosin low power and high power view insert at the right upper corner showing sheets of small blue cells with focal necrosis, high nuclear to cytoplasmic ratio, and focal areas of spindle cell formation. Immunohistochemical stains showing (B) CK CAM5.2 focally positive (C) WT1 focally positive and (D) Vimentin diffusely positive.

In-situ hybridization studies for kappa and lambda showed polytypic plasma cells. The submitted pathology slides revealed a hypercellular lesion with areas of epithelioid cytology; in other areas, they were rather spindled resembling sarcoma-like cells. A high nuclear to cytoplasmic ratio was noted with focal necrosis. A concern was raised for a biphasic tumor such as a metastatic carcinoma, malignant melanoma or mesothelioma but given the focal OSCAR keratin expression together with the morphology and the focal WT-1 expression in the absence of carcinoma markers, a malignant mesothelioma, biphasic type was diagnosed (Figure 3). 

Further workup with PET-CT scan was advised to evaluate the extent of the illness and cytotoxic chemotherapy combined with immunotherapy or tyrosine kinase inhibitors was recommended by oncology. The patient refused further imaging and treatment, and palliative care was consulted. 

## Discussion

MPM remains a rare form of cancer; however, the number of cases are increasing every year probably due to the spread of asbestos use. There is no established global baseline that can be used to evaluate the trend in disease occurrence [1]. The nonspecific clinical presentation of this condition can be misleading. Park, et al. have suggested that one mesothelioma case may be overlooked for every four to five recorded due to its insidious presentation [12]. Similar to our case, the patient had multiple visits to our institution with similar complaints before MPM was diagnosed. The final pathologic diagnosis can be also particularly difficult in cases of biphasic subtype, since the immunohistochemical test results might be discordant. Multiple biopsy samples and tissue sections are needed to demonstrate both cellular components [9]. Furthermore, extensive immunostaining is also necessary to diagnose MPM, which is fundamentally a diagnosis of exclusion. Histological morphology alone is not sufficient; positive and negative immunohistochemical markers are necessary to make a final diagnosis. New markers are emerging and are showing promising results; for example, glypican-1 immunohistochemistry has shown higher sensitivity and specificity as that of calretinin and D2-40 for epithelioid mesothelioma [13]. ALK-positive mesotheliomas have also been identified by immunohistochemistry in rare case and confirmed ALK rearrangement by fluorescence in situ hybridization [14]. The tumor described in our case had an unusual morphology, uncommon radiographic distribution and atypical immunohistochemical presentation.

The histologic subtype is still the best pathologic predictor of prognosis and pathologic staging is useful as a guide to surgical therapy. The Union for International Cancer Control and American Joint Committee on Cancer, Cancer Staging Manual is the most widely applied TNM system for evaluating the potential for tumor resectability; however, it is not a good predictor of prognosis [15]. Molecular prognostic factors like chromosomal alterations of the CDKN2A locus 9p21.3 in which a homozygous deletion by FISH is a marker of malignancy and poor prognosis with shorter survival and shorter time to relapse [16]. Germline BAP1 mutations, observed in 1%–2% of mesotheliomas appear to confer a favorable prognosis on the overall survival [17-18]. Likewise, immune checkpoint inhibitors have generated positive results for patients who have failed chemotherapy [10]. Novel immunotherapy is under investigation, a recent in vitro study evaluated afuresertib, showing a decreased survival of MPM cells by inhibiting the protein kinase Akt. This suggests that the catalytic, ATP-competitive Akt inhibition preferentially inhibits MPM cell growth compared with allosteric inhibitors such as perifosine [19].

## Conclusions

The insidious clinical presentation of MPM may cause a considerable delay in the final diagnosis along with a complex histology and atypical immunohistochemical characteristics. This may decrease survival especially if advanced stages with metastasis are present at the time of diagnosis. This neoplastic condition should be included in the differential diagnosis of elderly patients presenting with primary pleural mass with effusion, especially if there is a history of asbestos exposure. Physicians need to be aware that a thorough clinical history, high level of suspicion with extensive histological and immunohistochemical examination is required for definitive diagnosis.
